# Patent Ductus Arteriosus Occlusion in the Premature Infant: Persistent Research Gaps

**DOI:** 10.1016/j.jscai.2025.103878

**Published:** 2025-08-18

**Authors:** Frank F. Ing, Carl H. Backes

**Affiliations:** a Pediatric Heart Center, UC Davis Children’s Hospital, UC Davis School of Medicine, Sacramento, California; b Division of Neonatology, Department of Pediatrics, College of Medicine, The Ohio State University, The Heart Center, Nationwide Children’s Hospital, Columbus, Ohio

**Keywords:** congenital heart disease, patent ductus arteriosus, pediatrics, premature infant

In this issue of *JSCAI*, Gordon et al^1^ provide a Society for Cardiovascular Angiography & Interventions (SCAI) position statement on strategies to optimize the safety of transcatheter patent ductus arteriosus (PDA) occlusion among preterm infants. To ensure a broad and equitable perspective, the authors astutely assembled a multidisciplinary team of investigators. In the position statement, the authors underscore the importance of maintaining rigorous training and procedural competency standards for transcatheter ductal occlusion. They advocate for a multidisciplinary approach and set guidelines for proper patient selection using hemodynamic criteria to define a hemodynamically significant PDA. The investigators importantly call on the need for critical hospital infrastructure and dedicated services lines to optimize preprocedural and postprocedural care of these highly vulnerable infants. They also stressed the deleterious effects of a large PDA in a premature infant and compared closure rates between medical treatment, surgical ligation, and the transcatheter approach. Finally, the importance of regional coordination to ensure transcatheter ductal occlusion among extremely preterm infants (EPIs) is performed at institutions with the requisite expertise is emphasized.

While the authors should be commended for providing an evidence-based review of the available literature, fundamental questions remain unanswered prior to widespread adoption of transcatheter ductal occlusion. While there is promising data on safety and feasibility, evidence on the clinical efficacy of the procedure is lacking. To that end, no randomized controlled trials (RCTs) of transcatheter ductal occlusion vs alternative treatment strategies exist.

Despite considerable investigation for more than 7 decades, the optimal treatment for EPIs, (<28 weeks of gestation) with PDA remains elusive.^2^ Not surprisingly, PDA treatments vary widely among institutions and health care providers.^3^ Current technology has permitted occlusion in smaller patients, now as low as 700 g and even lower, in some experienced hands. In view of evidence showing that a persistent ductus is associated with greater rates of morbidity and mortality, some health care providers aggressively pursue treatment to ensure ductal closure. Alternatively, some health care providers have adopted a conservative (no definitive treatment) approach, citing evidence that all forms of deliberate ductal closure have potential risks, that many PDAs close spontaneously, and that no studies have shown that treating all EPIs with a PDA improves outcomes.^4^

Published nonrandomized data in neonatology journals show that a conservative approach in cases of prolonged PDA does not increase neurodevelopmental impairment or show significant differences in chronic lung disease, retinopathy of prematurity, necrotizing enterocolitis, severe intraventricular hemorrhage, mortality, or survival without morbidity.^5^^,^^6^ Meta-analyses and large-volume studies have shown substantial heterogeneity in patient profiles, timing of treatment, and inconsistency in outcome reporting, which underscores the complexity of PDA in premature infants, especially those who have extreme low birth weight (<1500 g).^3^^,^^7^, ^8^, ^9^

A summary of common agreements regarding the PDA and its treatment was published in 2019, which may serve as a template for further studies.^10^ A publication from the American Academy of Pediatrics on this topic stated that despite 6 decades of research on this topic, there is still “uncertainty and controversy about the significance, evaluation, and management of the PDA in preterm infants resulting in heterogeneity in clinical practice.”^2^ This article concluded that there remain research gaps in the benefits and risks of various management strategies beyond the first 2 weeks of life, the long-term cardiopulmonary and neurodevelopmental outcomes, and the need for a well-designed trial to answer these questions. Fast forward to the present, these gaps remain.^11^

It is generally accepted that a subpopulation benefits from closing a PDA, but the cohort and timing of treatment is not well studied. The hemodynamically significant PDA is not as straightforward as described by the authors of the position statement for several reasons as follows:

1. While a PDA in a full-term infant or older is abnormal, this is often viewed as normal in premature infants due to prematurity and because most spontaneously close and can be managed conservatively.
2. Successful neonatal management of the premature population has improved to permit survival of more premature (as low as 22-week gestation) and lower birth weight (as low as 400 g) infants, resulting in new clinical challenges.
3. Despite straightforward hemodynamic abnormalities caused by the left-to-right shunting of the PDA, actual cause-and-effect relationships to other organ systems (eg, lungs, kidneys, brain, and gut) are not consistent in the premature infant.
4. Treatment modalities (medications, surgical ligation, or transcatheter closure) in the premature population have evolved significantly over the past 3 decades, and previous comparative studies may not be relevant to current realities.
5. Timing to treatment as related to degree of prematurity have not been studied adequately.
6. Short-term improvements do not necessarily reflect long-term outcomes (ie, comparison of neurodevelopmental outcomes has not shown superiority for PDA closure by either surgery or transcatheter techniques vs conservative treatment).

Until robust evidence is accumulated, the present position statement thoughtfully provides a roadmap for health care providers. To answer fundamental questions on the optimal timing and use of transcatheter ductal closure among EPIs, equipoise among health care providers in the enrollment of their patients into randomized controlled trials is needed.^8^ Because of entrenched biases and beliefs regarding best PDA treatment practices, lack of physician equipoise in previous multicenter PDA trials has precluded patient enrollment and limited generalizability of findings.^12^

The interventional pediatric cardiology community should be commended for designing devices that address the unique ductal morphology of the EPI, adopting procedural modifications (eg, avoidance of arterial access) that minimize risk, and leading prospective, nonrandomized trials that inform health care providers regarding the safety and feasibility profiles of the procedure.^13^^,^^14^ Unlike other congenital heart disease, the PDA in the premature infant is not managed by pediatric cardiologists, nor are they the gatekeepers of this lesion. The neonatologists are the primary gatekeepers, and it is now time for neonatal community to take the important next step: The design of contemporary, pragmatic RCTs that addresses unanswered questions on the clinical efficacy of the procedure. These trials must include short-term and long-term outcomes that are meaningful not only to health care providers but also to the families we aim to serve. In view of recent data showing increased harm from PDA drug therapy administered in the first postnatal weeks, uncertainty remains regarding evidence-based approaches for EPIs with an open, hemodynamically significant PDA beyond the first 2 weeks of life.^15^ Hence, in the absence of robust, RCT-level evidence, health care providers are left with insufficient data to guide the practice of evidence-based medicine.^5^

For more than 7 decades, the neonatal community have cited evidence from observational studies to highlight the association between the presence of a PDA and adverse outcomes.^16^ Now equipped with a definitive treatment option to close the PDA, can the neonatal community work together to successfully execute a pragmatic RCT that sheds light on the potential causal effect of a persistent hemodynamically significant PDA on important neonatal outcomes? Pediatric interventional cardiologists understand the hemodynamic effects of a large PDA, a knowledge passed down through generations of pediatric cardiologists, who often favor eliminating this lesion, assuming it can only benefit other body systems that might be negatively affected. However, in extremely premature infants with low birth weight—who are primarily managed by neonatologists and whose physiology may not exactly follow that of full-term or older infants—there is still a need for trials. These studies are essential to better define when, how, and whom to treat for PDA, filling research gaps before establishing interventional guidelines. The pediatric cardiologists have done their part—now the responsibility shifts to the neonatologists to fulfill their role.

## Peer review statement

Associate Editor Frank F. Ing had no involvement in the peer review of this article and has no access to information regarding its peer review. Full responsibility for the editorial process for this article was delegated to Editor-in-Chief Alexandra J. Lansky.
